# Cathepsin D mediates tachykinin-induced secondary follicle growth independent of the hypothalamic–pituitary–gonadal axis in mice

**DOI:** 10.3389/fendo.2025.1621348

**Published:** 2025-06-05

**Authors:** Tsuyoshi Kawada, Masato Aoyama, Keiko Yasuda, Honoo Satake

**Affiliations:** ^1^ Bioorganic Research Institute, Suntory Foundation for Life Sciences, Kyoto, Japan; ^2^ Department of Chemistry, Biology, and Environmental Science, Faculty of Science, Nara Women’s University, Nara, Japan

**Keywords:** tachykinin, cathepsin D, follicle growth, immature follicle, granulosa cell

## Abstract

**Background:**

Cathepsin D is an aspartic protease responsible for the proteolytic processing of vitellogenin at the early stages of folliculogenesis in oviparous vertebrates. Previously, we identified a multifunctional neuropeptide, tachykinin (TK), as an inducer of cathepsin D that promotes vitellogenic follicle growth in the ascidian *Ciona intestinalis type A* (*Ciona robusta*), a sister group of vertebrates. However, no regulatory factor for cathepsin D in the ovary has been identified in vertebrates. Moreover, the involvement of cathepsin D in mammalian folliculogenesis has yet to be investigated.

**Methods:**

Ovaries of 2-week-old ICR mice were used. *Cathepsin D* gene expression in the ovaries was examined by real-time PCR. Localization of cathepsin D was shown by immunostaining of ovarian sections. Cathepsin D activity was measured using supernatants from the homogenized ovaries. Mouse follicle growth was evaluated using three-dimensional follicle culture system.

**Results:**

Immunohistochemical analysis revealed that cathepsin D is co-localized with TK receptors in granulosa cells of secondary follicles in the ovaries of two-week-old mice, which are sexually immature and in which the hypothalamus–pituitary–gonadal (HPG) axis is not yet functional. TK treatment of the ovaries significantly increased *cathepsin D* gene expression and its proteolytic activity. Moreover, inhibition of cathepsin D markedly suppressed the secondary follicle growth.

**Conclusion:**

Collectively, these results indicate that cathepsin D plays essential roles in mouse secondary follicle growth. Recently, we also demonstrated that ovarian TKs promote secondary follicle growth in mice, primarily in a paracrine/autocrine manner. Combined with these findings, the present study leads us to propose an evolutionary scenario in which TK–cathepsin D signaling functions as a conserved mechanism for HPG axis-independent follicle growth across chordates, which may be more broadly conserved than the vertebrate-specific, HPG axis-dependent systems.

## 1 Introduction

In mammals, follicular development progresses through a well-defined sequence of stages: primordial, primary, secondary, pre-antral, antral, and Graafian follicles (1–5). This process is largely classified into gonadotropin-independent and gonadotropin-dependent phases. In postpubertal females, follicle-stimulating hormone (FSH) and luteinizing hormone (LH), secreted from the anterior pituitary in response to hypothalamic gonadotropin-releasing hormone (GnRH), play essential roles in promoting the growth and maturation of pre-antral to antral follicles through the hypothalamus–pituitary–gonadal (HPG) axis. In contrast, the growth of early-stage follicles—including primordial, primary, and secondary follicles—is independent of gonadotropins and occurs prior to the onset of HPG axis functions (6, 7).

Tachykinins (TKs) are a conserved family of neuropeptides found across chordates, including ascidians. In mammals, TKs function in diverse physiological contexts, acting as neuromodulators and peripheral hormones that regulate pain, inflammation, gastrointestinal motility, immune response, hematopoiesis, and reproductive processes (8–10). Mammalian TKs include substance P (SP), neurokinin A (NKA), neurokinin B (NKB), and hemokinin-1/endokinins (HK-1/EKs), which exert their effects through specific G-protein-coupled receptors: TACR1, TACR2, and TACR3 (8, 11–13). Our previous study demonstrated that ovarian TKs promote secondary follicle growth in mice, primarily through autocrine/paracrine signaling (14). TKs stimulate the expression of cyclooxygenase-2 (COX-2) via the JAK1–STAT3 pathway in granulosa cells, resulting in elevated production of prostaglandins E2 (PGE2) and F2α (PGF2α). Moreover, we previously demonstrated that TK specifically triggered the growth of vitellogenic follicles via induction of the expression of several proteases, including cathepsin D, in the ascidian *Ciona intestinalis type A* (*Ciona robusta*), a sister group of vertebrates (15, 16). These findings verified that cathepsin D acts as a direct downstream effector of TK signaling for early-stage follicles in *Ciona*.

Cathepsin D is a lysosomal aspartic protease widely conserved across metazoans (17–19), and plays vital roles in protein turnover, activation of hormones and growth factors, enzymatic processing, and apoptosis. In oviparous vertebrates, cathepsin D is known to facilitate the breakdown of yolk precursors such as vitellogenin, thereby providing energy for oocyte growth (20–22). Despite these known functions, the physiological role and regulatory mechanisms of cathepsin D in mammalian folliculogenesis remain largely unexplored.

In this study, we revealed the involvement of TK-induced cathepsin D in secondary follicle development in mice, and provides new insight into a conserved HPG axis-independent mechanism of early-stage folliculogenesis.

## 2 Materials and methods

### 2.1 Animals

This study was approved by the Suntory animal ethics committee and the Animal Care Committee of Nara Women’s University, and all animals were maintained in accordance with committee guidelines for the care and use of laboratory animals. This study was approved by the Suntory animal ethics committee (APRV000340). ICR mice were purchased from Japan SLC Inc (Kyoto, Japan). All of the mice were euthanized with CO_2_ asphyxiation.

### 2.2 Immunohistochemistry

2-week-old mouse ovaries were fixed at 25°C for 15 min in Bouin fluid. The fixed ovaries were embedded in paraffin, and the ovaries were cut into 7-µm sections. Immunostaining using primary antibodies (CathepsinD(R-20) and NK-1R(H-83); Santa Cruz Biotechnology, CA, USA) was performed as previously described (14). The immunoreactivity was visualized with an indirect immunofluorescence technique using secondary antibodies (Alexa 488 donkey anti-rabbit IgG and alexa 568 donkey anti-goat IgG; Life Technology, Carlsbad, CA, USA) diluted with blocking buffer (1:500; v/v). Coverslips were mounted in Fluorosafe mounting medium (Merck, Darmstadt, Germany), and the sections of ovaries were viewed using an Olympus BX51 photomicroscope (Olympus, Tokyo, Japan) equipped with epifluorescence. Because auto-fluorescence was detected in the ovarian sections, a WIB long-pass filter cube (Olympus) was used for observation of cathepsin D-derived fluorescent signals.

### 2.3 Real-time PCR

The real-time PCR was performed using CFX96 Real-time System and SsoAdvancedTM Universal SYBR Green Supermix (Bio-Rad laboratories, Hercules, CA, USA). Total volume of the real-time PCR reaction mixture was 20 µl consisting of 100 ng template cDNA, each 500 nM primer, and 10 µl SsoAdvancedTM Universal SYBR Green Supermix. The real-time PCR program was at 95°C for 30 sec, and 44 cycles of at 95°C for 15 sec and at 60°C for 30 sec. The melting curve analyses for amplified PCR products were performed to confirm the absence of primer dimers. To evaluate the gene expressional level, we analyzed using ΔΔCt method that represents the induction level of target genes in the ovaries treated with TACR1, 2, and 3 agonists or antagonists (**Supplementary Table 1**). Ct value represents the PCR cycle number when the PCR product is arrived at determined level, and ΔCt shows the difference between Ct values using PCR products prepared from the agonists-treated and the antagonists-treated ovaries. Subsequently, ΔΔCt was calculated using ΔCt of *β-actin* gene between the agonists-treated and antagonists-treated ovaries to normalize the real-time PCR result. The primers used for the real-time PCR were designed using Primer-blast web tool (https://www.ncbi.nlm.nih.gov/tools/primer-blast/), and their sequences are shown in **Supplementary Table 2**.

### 2.4 Measurement of cathepsin D activity in the ovary

The half-piece ovaries were incubated in the presence of TACR1, 2, and 3 agonists or antagonists (**Supplementary Table 1**). The agonist-treated or antagonist-treated ovaries were homogenized in Cathepsin D assay buffer included in the Cathepsin D Assay Kit (Sigma-Aldrich, St. Louis, MO, USA, for the Cathepsin D assay), and centrifuged at 15000 x g at 4°C for 5 min. The supernatants were frozen with liquid nitrogen, and stored at -80°C until use. The cathepsin D activity was evaluated using the Cathepsin D Assay Kit (Sigma-Aldrich) according to the manufacturer’s instruction. The difference of cathepsin D activities between agonist-treated and antagonist-treated ovaries was calculated.

### 2.5 Three-dimensional follicle culture system

Co-cultivation of mouse secondary follicles with theca/interstitial cells using collagen gel (Cellmatrix Type I-A; Nitta Gelatin, Inc., Osaka, Japan) was performed as described in previous reports (23, 24). In brief, 0.2% collagen gel containing 10% FBS (Thermo Fisher Scientific Inc.), 100 U/ml penicillin, 0.1 mg/ml streptomycin (Nacalai Tesque Inc., Kyoto, Japan), and Dulbecco’s Modified Eagle Medium components (Nissui Pharmaceutical Co., Ltd., Tokyo, Japan) were used to culture the secondary follicles. Approximately 20–30 secondary follicles with 100 μm-diameter and 6 × 10^4^/well-theca/interstitial cells were co-cultured at 37°C in 5% CO_2_ in air and 100% humidity for 5 days in a 96-well plate with no ligand or pepstatin A as a cathepsin D inhibitor. Morphological change of each follicle was observed using an inverted microscopy Olympus CK2 (Olympus). Short and long diameters of oocytes and follicles were measured using an image analysis tool, ImageJ (https://imagej.nih.gov/ij/). Oocyte and follicle growth were calculated differential length of diameters between oocytes and follicles culturing for 0 day and 5 days.

### 2.6 Statistical analysis

Results are shown as mean ± S.E.M. Data were analyzed by t test with Welch’s correction or one-way ANOVA and turkey’s multiple comparison test. Differences were accepted as significant for P < 0.05.

## 3 Results

### 3.1 Localization of cathepsin D in the mouse ovary

**Figure 1:**
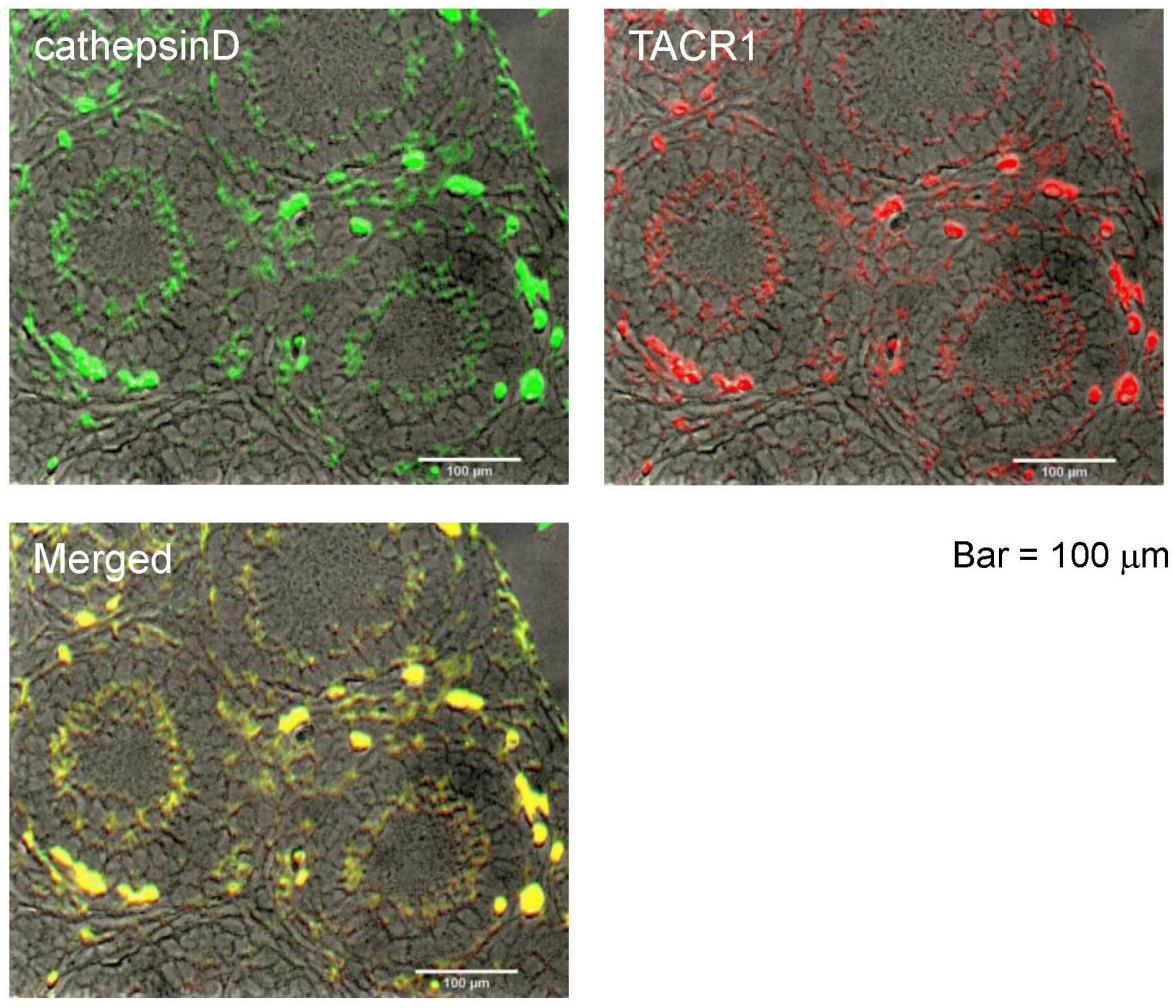
Localization of cathepsin D in the ovaries of 2-weeks old mouse. Immunostaining of cathepsin D on a section of the ovary is shown by green signal, while immunostaining of TACR1 is shown by red signal. Yellow signal in the merged image shows the co-localization of cathepsin D and TACR1. Scale bars indicate 100 μm.

All TK receptors (TACR1, -2, and -3), are found to be colocalized in the granulosa cells of the inner layer within secondary follicles, and their activation triggers the growth of these follicles (14). Immunostaining of sections from 2-week-old mouse ovaries revealed that cathepsin D is localized in the granulosa cells of the inner layer of secondary follicles, showing strong spatial correspondence with the immunoreactivity of TACR1 (**Figure 1**). Additionally, TACR1 was shown to be co-localized with both TACR2 and 3 in the granulosa cells of the inner layer of secondary follicles in our previous study (14), indicating that cathepsin D is co-localized with all three TACRs.

### 3.2 Downregulation of *cathepsin D* gene expression and activity by TK receptor antagonists

**Figure 2:**
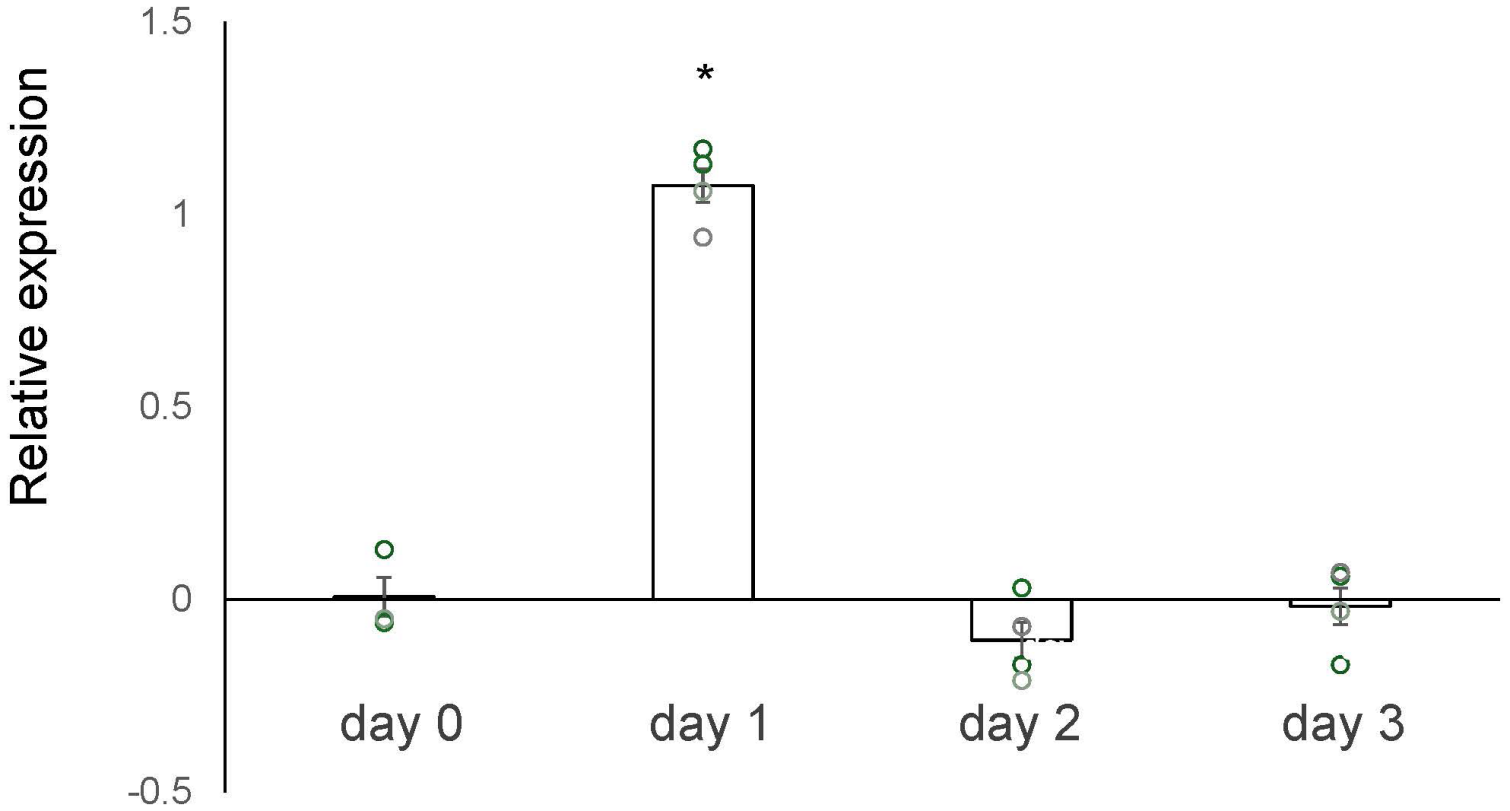
Real-time PCR-based quantification of the *cathepsin D* gene expression. The vertical axis represents the relative level of the *cathepsin D* gene expression in the ovary treated with TK receptor agonists (each agonist at 1 μM), compared to that treated with TK receptor antagonists (each antagonist at 1 μM). The induction level of gene expression was calculated from the ΔΔCt values using *β actin* gene in the condition of presence of TK receptor agonists or TK receptor antagonists. Each point represents the mean ± S.E.M for three or four independent experiments. Significant differences (P< 0.05 *vs*. TK antagonist-treated group with t-test) are indicated by asterisks.

**Figure 3:**
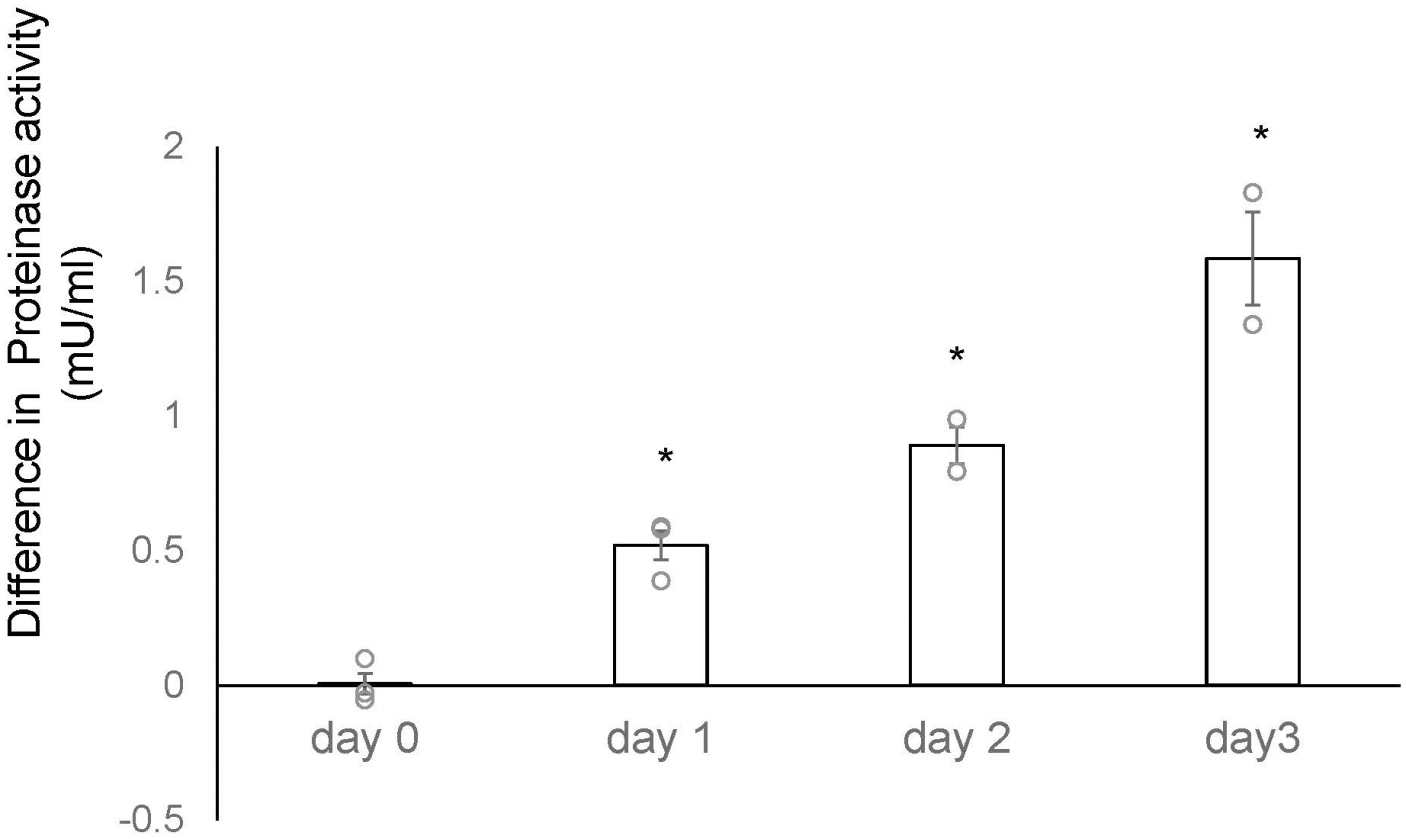
Time-course analysis of differences in cathepsin D activity between 2-week-old mouse ovaries treated with TK receptor agonists and those with antagonists. The vertical axis represents the difference in proteinase activity between ovaries cultured with TK receptor agonists and those cultured with TK receptor antagonists. Cathepsin D activity was gradually upregulated in the ovary treated with TK receptor agonists (each agonist at 1 μM) compared to those treated with TK receptor antagonists (each antagonist at 1 μM). Each point represents the mean ± S.E.M for two independent experiments. Significant differences (P< 0.05 *vs*. TK receptor antagonist-treated group with t-test) are indicated by asterisks.

We conducted a microarray analysis on 2-week-old mouse ovaries treated with either TK receptor agonists or antagonists to identify factors induced by TK (14; accession no. GSE213246). The microarray data suggested that the *cathepsin D* gene was also downregulated in ovaries treated with TK receptor antagonists. To verify the changes in *cathepsin D* gene expression, we performed real-time PCR on cDNAs prepared from the 2-week-old mouse ovaries treated with either TK receptor agonists or antagonists. Real-time PCR revealed a 2.1-fold increase in *cathepsin D* gene expression in ovaries treated with TK receptor agonists for one day, compared to those treated with antagonists (**Figure 2**). However, after more than two days, the expression levels of the *cathepsin D* gene were comparable between the ovaries treated with agonists and those treated with antagonists (**Figure 2**, **Supplementary Table 3**). These data indicated that the activation of TK receptors transiently upregulated *cathepsin D* gene expression, which was rapidly downregulated thereafter. Subsequently, we assessed the protease activity of cathepsin D in the 2-week-old mouse ovaries treated with TK receptor agonists or antagonists. These assays indicated that ovaries treated with TK receptor agonists exhibited higher protease activity compared to those treated with antagonists (**Figure 3**, **Supplementary Table 4**), proving that TKs stimulate the production of cathepsin D in the ovaries. Notably, cathepsin D activity remained consistently higher in ovaries treated with TK receptor agonists than in those treated with antagonists, despite comparable levels of *cathepsin D* gene expression in both groups after more than two days. Together with the immunostaining results, these findings suggest that the increase in cathepsin D activity is directly triggered by TK receptor activation in granulosa cells of the inner layer of secondary follicles.

### 3.3 Suppression of follicle growth by cathepsin D inhibitor

**Figure 4:**
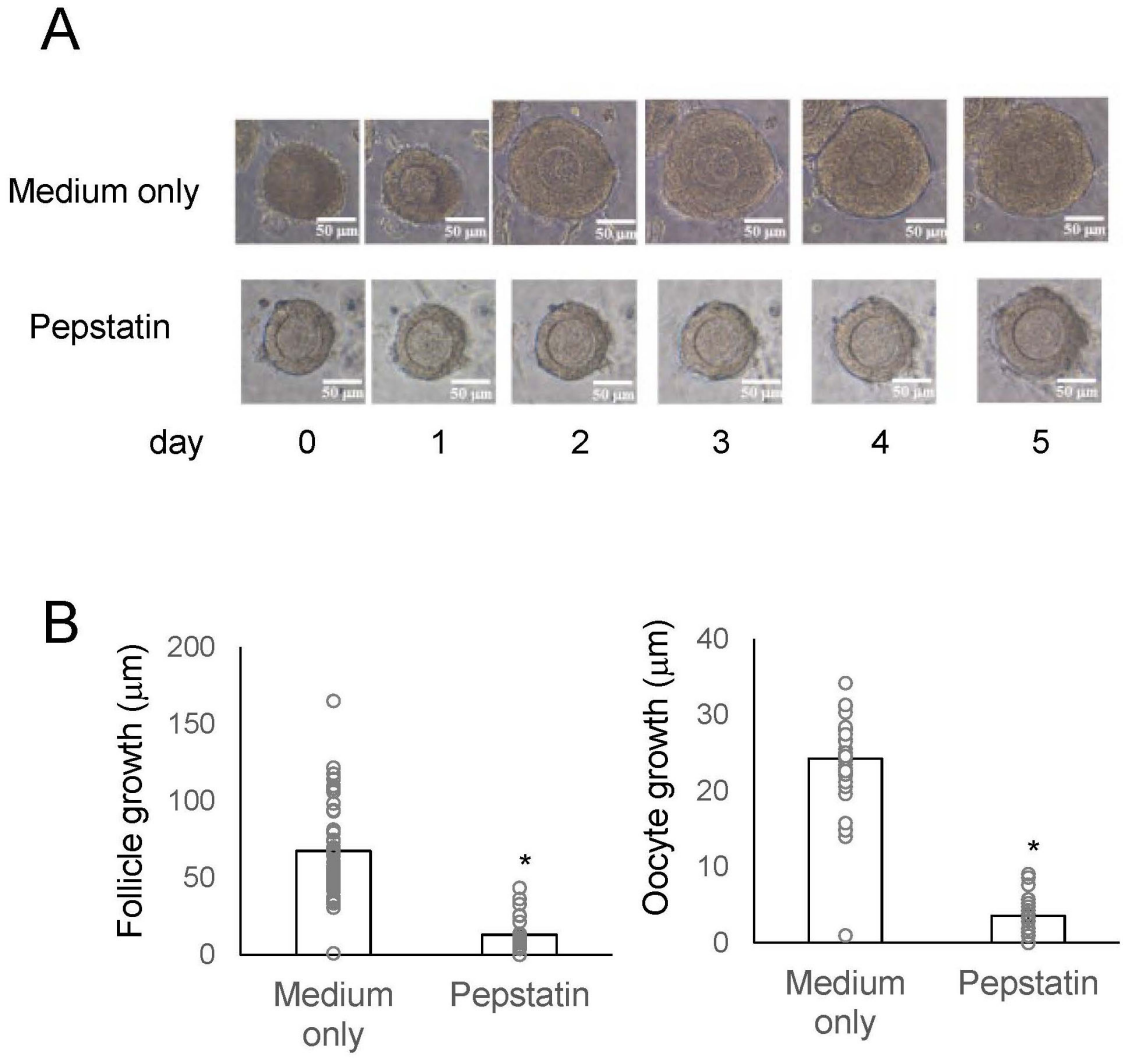
Effects of a cathepsin D inhibitor on secondary follicle growth in three-dimensional follicle culture. **(A)** Secondary follicles isolated from the mouse ovary were cultured within a collagen gel with theca cells for 5 days. The follicles were untreated (medium only) or treated with 8 μg/ml-pepstatin A (cathepsin D inhibitor). Bars indicate 50 μm. **(B)** The size of the follicles grown in three-dimensional culture in the presence or absence of 8 μg/ml-pepstatin for 5 days was measured. Oocyte growth is represented as the increase in oocyte diameter from Day 0 to Day 5, calculated by subtracting the diameter at Day 0 from that at Day 5. Likewise, follicle growth is represented as the increase in follicle diameter over the same period. Each point represents the mean ± S.E.M. Significant differences (P< 0.05 *vs*. medium only group with t-test) are indicated by asterisks.

Subsequently, we evaluated the impact of cathepsin D on secondary follicle growth by *in vitro* morphological assays using three-dimensional follicle culture (14, 23, 24). We utilized an aspartic protease, pepstatin A, as a cathepsin D inhibitor. Of particular interest is that the follicle growth was suppressed by approximately 80% in the presence of pepstatin A, while normal growth occurred in its absence (**Figure 4**). Similarly, the growth of oocytes within the secondary follicle was also obstructed by pepstatin A (**Figure 4**). The inhibitory effect of pepstatin A on cathepsin D activity indicates that cathepsin D is critical for secondary follicle growth, such as the role of TKs. Overall, these results verified that mouse TKs induce the secondary follicle growth via the upregulation of the gene expression and enzymatic activity of cathepsin D in the mouse ovary.

## 4 Discussion

Our current study reveals a novel role for cathepsin D in the growth of mouse secondary follicles. Cathepsin D promotes follicle growth through the proteolysis of vitellogenin, a process previously observed in various oviparous vertebrates and invertebrates (20, 21, 25, 26). Vitellogenin serves as a precursor protein of egg yolk, which is stored in oocytes and utilized as a nutrient source during egg laying and early embryonic development. In contrast, the *vitellogenin* gene has been lost in viviparous species during the mammalian evolution (27), indicating that in viviparous organisms, cathepsin D may contribute to reproductive functions beyond yolk protein processing. Cathepsin D is also known to play a vital role in maintaining cellular homeostasis. In humans, it regulates protein turnover by degrading misfolded and aggregated proteins and facilitates apoptosis under conditions of proteostatic stress (19). In mouse follicles, granulosa cells undergo substantial proliferation as follicles develop from the secondary to pre-antral stage, necessitating quality control and remodeling for normal growth. It is therefore plausible that cathepsin D contributes to these processes during follicular development in mice.

Recent advancements of follicle culture systems *in vitro* have been significant, with mature follicles successfully generated from pluripotent stem cells in mice (28, 29). The culture medium used for follicle maturation includes factors such as GDF9, BMP15, FSH, EGF, and hCG, all of which are essential contributors to the development of mature follicles. Nevertheless, the molecular mechanisms in each cell that comprising the follicle during its development and maturation are not yet well characterized. As follicles develop, granulosa cells undergo processes of formation, proliferation, and multilayering, with specialized subtypes such as cumulus cells emerging. These granulosa cells are believed to shift their functions across both temporal and spatial dimensions. It has been proposed that TK-cathepsin D signaling specifically contributes to the proliferation and stratification of granulosa cells within secondary follicles. In the future, the increasing use of single-cell transcriptomic technologies is expected to shed light on the distinct temporal and spatial functions of individual granulosa cell populations.

Interestingly, the timing of *cathepsin D* gene upregulation in mouse secondary follicles does not align with the peak of its protease activity (**Figures 2**, **3**). Cathepsin D is initially synthesized as pre-pro-cathepsin D from its mRNA (20, 21, 25, 26). Following the removal of the signal peptide, pro-cathepsin D is transported into intracellular vesicles, where it undergoes proteolytic maturation into the active form through the action of cysteine proteases and other cathepsins (e.g., cathepsins B and L) within endosomes and lysosomes (17–19). This post-translational processing is in a good agreement with the observed gap between *cathepsin D* gene expression and its enzymatic activity. Consistently, in *Drosophila* ovaries, *cathepsin D* gene expression is elevated during vitellogenesis, whereas mature cathepsin D protein accumulates to higher levels during follicular atresia (21). Eventually, the protease activity is retained in the ovaries during atresia, despite lower gene expression levels (21). These findings support the notion that in mouse secondary follicles, cathepsin D activity in granulosa cells is regulated not only at the transcriptional level but also through precursor processing at the post-translational level.

**Figure 5:**
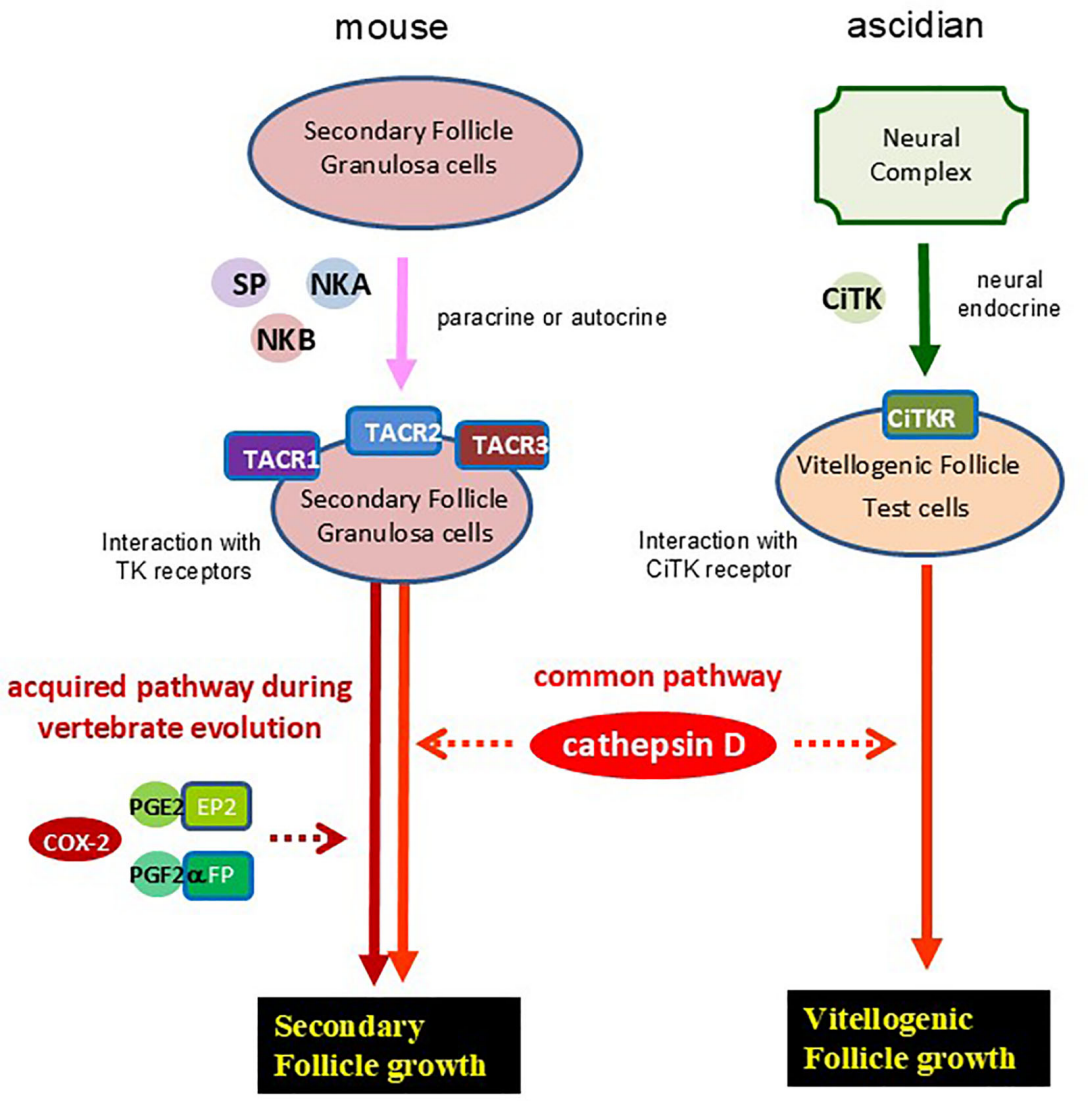
An evolutionary hypothesis of TKergic follicle growth in chordates. Mouse TKs (SP, NKA, and NKB) are secreted from granulosa cells of secondary follicles, and selectively interact with TK receptors (TACR1, -2, and -3) at the granulosa cells with autocrine or paracrine. Activation of TK receptors induces secondary follicle growth, which is mediated by cathepsin D activation and interaction between prostaglandins (PGE2 and PGF2α) and their receptors (EP2 and FP, respectively) (14). On the other hand, *Ciona* TK (CiTK) is secreted from the ascidian neural complex, and is secreted to the ovary via a neuroendocrine pathway. Activation of CiTK receptor (CiTKR) induces the *cathepsin D* gene expression, leading to the vitellogenic follicle growth in *Ciona* (15, 16).

Ascidians are aquatic organisms found globally, classified within the phylum Urochordata and the superphylum Chordata, and are among the closest relatives of vertebrates (30–32). In *Ciona*, cathepsin D is localized in the test cells that surround oocytes within vitellogenic follicles (15, 16, 33, 34). The geometric arrangement of these test cells closely resembles that of granulosa cells surrounding oocytes in vertebrate follicles (35), suggesting that several functional characteristics are at least in part conserved between ascidian test cells and vertebrate granulosa cells (**Figure 5**). Notably, *Ciona* TK (CiTK) promotes the growth of vitellogenic follicles in *Ciona* through an increase in cathepsin D levels (16). Combined with these findings, the present study suggests that the essential mechanism underlying TK-cathepsin D-mediated early-stage follicle growth is essentially conserved throughout chordates and have originated at least in a common ancestor of vertebrates and urochordates (**Figure 5**). Conversely, the regulatory mechanisms governing TK–cathepsin-mediated follicular development differ between the mouse and ascidian. In the ascidian, CiTK is secreted from the central nervous system, reflecting a “centralized” neuroendocrine regulation. In contrast, in the mouse ovary, TKs are synthesized within the follicle and act through a “locally autonomous” paracrine or autocrine signaling system. Consequently, it is postulated that this locally autonomous TKergic system emerged alongside the evolutionary advancement and functional specialization of vertebrate tissues.

Recently, we demonstrated that PGE2 and PGF2α are crucial for the growth of TK-mediated secondary follicles in mice (14). TKs induce the upregulation of the *COX-2* gene, which encodes prostaglandin H2 (PGH2) synthase, in mouse ovaries, and increase of PGH2 synthesis leads to the production of PGE2 and PGF2α (14). Combined with these findings, the present study indicates that TKs facilitate the growth of secondary follicles in mice through both prostaglandin and cathepsin D signaling pathways. In contrast, the previous gene expression profile revealed that the *COX-2* gene, unlike the *cathepsin D* gene, was not upregulated by treatment of *Ciona* ovaries with CiTK. These results indicate that the TK-cathepsin D signaling pathway involved in early follicular development is evolutionarily conserved across chordates, whereas the TK-COX-2 signaling pathway has been acquired by limited chordates. Likewise, the HPG axis, a key regulatory system of follicle growth, is restricted to vertebrates. Thus, the TK-cathepsin D system is likely to be more broadly conserved, compared with both the TK-COX-2 system and the HPG axis. In contrast, the substrate of cathepsin D in the TKergic follicle growth is not conserved among chordates. In viviparous species, developing embryos receive nutrients directly from the maternal body, which diminishes the necessity for substantial yolk reserves within ovarian follicles. This shift in reproductive strategy might have reduced the functional relevance of yolk-associated proteins, such as vitellogenin, during follicle development. Consequently, it is hypothesized that, during the evolutionary transition from oviparous to viviparous species, the cathepsin D substrate in TKergic follicle growth shifted from vitellogenin to other proteins.

Our previous study demonstrated that the JAK1–STAT3 signaling promotes *COX-2* gene expression in granulosa cells of mouse secondary follicles (14). In contrast, the signaling pathway responsible for inducing *cathepsin D* gene expression in these cells remains unidentified. Indeed, the functional relationship between the TK-COX-2 signaling and TK-cathepsin D signaling has not been found in any animals. In MCF−7 human breast cancer cells, the *cathepsin D* gene expression is regulated by multiple pathways. Estrogen directly induces cathepsin D expression through estrogen-responsive elements in the gene promoter region (36, 37), while growth factors like IGF-I, TGFα, and EGF also elevate cathepsin D level via alternative mechanisms, including MAPK-mediated activation of estrogen receptor and Sp1 binding to GC-rich promoter regions in MCF-7 cells (37). Nevertheless, the signaling pathways for regulation of *cathepsin D* gene expression remain poorly characterized in most other cell types and tissues. Future studies will aim to elucidate the TK–cathepsin D signaling pathway in granulosa cells of mouse secondary follicles in greater detail.

## 5 Conclusion

We have provided original evidence for a secondary follicle growth process mediated by the TKergic cathepsin D activation. Our findings will enhance the understanding of not only gonadotropin-independent follicle growth mechanisms in mammals but also the evolutionary pathways of follicle growth systems throughout chordates.

## Data Availability

The datasets presented in this study can be found in online repositories. The names of the repository/repositories and accession number(s) can be found in the article/**Supplementary Material**.
