# Supplementary material for: Cathepsin D mediates tachykinin-induced secondary follicle growth independent of the hypothalamic–pituitary–gonadal axis in mice
Source: Front Endocrinol (Lausanne). 2025 Jun 5;16:1621348. doi: 10.3389/fendo.2025.1621348 (PMC12176580; doi:10.3389/fendo.2025.1621348)
Supplement: Supplementary file 1 [file Table1.docx]

Supplementary Material

**Supplementary Table 1.** List of tachykinin receptor ligands

| Source | agonists or antagonists | Description (target) |
| --- | --- | --- |
| Sigma‒Aldrich | [Sar9,Met(O_2_)11]-SP | TACR1 agonist |
| Sigma‒Aldrich | GR-64349 | TACR2 agonist |
| Sigma‒Aldrich | Succinyl-[Asp, N-Me-phe8]sp.6-11 | TACR3 agonist |
| Sigma‒Aldrich | L-703,606 | TACR1 antagonist |
| Sigma‒Aldrich | GR-94800 | TACR2 antagonist |
| Sigma‒Aldrich | SB218795 | TACR3 antagonist |

**Supplementary Table 2.** List of primers for Real-time PCR

| GenBank refseq | gene | FW primer | RV primer |
| --- | --- | --- | --- |
| NM_009983 | *cathepsin D* | caagcagcctggaatcgtattt | gaccggaagcacgttgttaac |
| NM_007393 | *β-actin* | ccgtgaaaagatgacccagatc | cacagcctggatggctacgt |

**Supplementary Table 3.** ΔΔCt value for Real-time PCR of *cathepsin D* gene

| gene | incubation days | upregulation by TK receptor agonists |
| --- | --- | --- |
| *cathepsin D* | 0  1  2  3 | 2^-ΔΔCt^ = 0.007 ± 0.05  2^-ΔΔCt^ = 1.08 ± 0.04  2^-ΔΔCt^ = -0.11 ± 0.05  2^-ΔΔCt^ = 0.02 ± 0.05 |

**Supplementary Table 4.** Proteinase activity of ovarian extract.

| treated ligands | day 0 | day 1 | day 2 | day 3 |
| --- | --- | --- | --- | --- |
| TK receptor agonists | 0.32 ± 0.04 | 0.92 ± 0.07 | 2.72 ± 0.06 | 3.66 ± 0.27 |
| TK receptor antagonists | 0.30 ± 0.02 | 0.40 ± 0.01 | 1.83 ± 0.04 | 2.07 ± 0.02 |

mU / ml
